# Exploring the Effect of a Wavy Sea Surface on NLOS-UOWC Systems: A Novel Deterministic Approach

**DOI:** 10.3390/s25030695

**Published:** 2025-01-24

**Authors:** Paulo Samaniego-Rojas, Rubén Boluda-Ruiz, José María Garrido-Balsells, Beatriz Castillo-Vázquez, Antonio Puerta-Notario, Antonio García-Zambrana

**Affiliations:** Telecommunication Research Institute (TELMA), Universidad de Málaga, E-29010 Málaga, Spain; rbr@ic.uma.es (R.B.-R.); jmgb@ic.uma.es (J.M.G.-B.); bcv@ic.uma.es (B.C.-V.); apn@ic.uma.es (A.P.-N.); agz@ic.uma.es (A.G.-Z.)

**Keywords:** underwater optical wireless communication, non-line of sight, channel modeling, wavy surface

## Abstract

This work presents a novel approach to modeling an underwater optical wireless communications (UOWC) channel based on a deterministic analysis specifically for non-line-of-sight (NLOS) configurations. The model considers the presence of a wavy ocean surface, providing a more accurate representation of realistic conditions. By expanding the possibilities for communication in complex underwater environments, our model offers a comprehensive analysis of the ocean waves’ impact. A significant achievement of this study is the capacity of the model to accurately compute the variable size of the width of the beam (footprint) on the receiver plane reflected by the sea surface and the time intervals during which the receiver remains illuminated. Additionally, the model determines the precise position of the reflected beam on the receiver plane and accurately identifies the time intervals during which communication is feasible, offering invaluable insight into the system performance under oceanic wave variability. The results confirmed that oceanic wave variability induces severe misalignment in optical links, creating intermittent opportunities for effective communication. The optical–geometric analysis contributed significantly to understanding the novel impact of ocean waves on NLOS-UOWC systems. These findings enhance the preliminary considerations in NLOS link design, particularly in scenarios with autonomous underwater vehicles in constant motion, aiding in the reduction of pointing errors.

## 1. Introduction

Technological advances in oceanography are not limited to natural sciences and related disciplines but are also evident in novel navigation, communication, and exploration systems. These advances enable the maritime sector to conduct extensive scientific research and operational activities in the sea and oceans, addressing critical challenges and opportunities. Some of their main motivations are the search for resources such as oil and gas, the early prediction of natural disasters, and promoting the care of the environment using underwater sensor networks. However, the importance of advancing submarine communications technology goes beyond environmental preservation. Inter-oceanic communications is another rapidly expanding field that demands high-performance submarine technology, currently supported by a vast undersea infrastructure of cables and offshore data centers. Traditional underwater wireless communication methods, mainly acoustic, have seen significant advancements but still face notable challenges, such as a limited bandwidth and high latency, despite their long-range capabilities [1]. In contrast, optical wireless technology is emerging as a promising alternative for the development of underwater optical wireless communication (UOWC) technology. This technology is notable for its ability to provide high data rates, improved security, a small beam footprint, and low latency [2]. UOWC links can be configured in two primary ways: line of sight (LOS) and non-line of sight (NLOS). LOS links, which require a direct path between the transmitter and receiver, have received considerable attention from the scientific community due to their high efficiency and reliability in controlled conditions. Despite this focus, NLOS links hold significant potential for addressing challenges in complex underwater environments that can hinder the effectiveness of LOS point-to-point connections. So, various factors, such as marine flora and fauna, complex underwater bathymetry, and other potential natural and artificial factors, can block the propagation of the optical beam in underwater environments. In practical application scenarios, these obstructions pose significant challenges to establishing reliable line-of-sight (LOS) links, particularly in scenarios involving dynamic underwater conditions. In this way, NLOS links emerge as a feasible option to overcome these obstacles by using reflections at the interface between the ocean and air [3]. Thus, as mentioned above, some of the applications of this technology are related to scenarios where data transmission is performed between devices, such as from autonomous underwater vehicles (AUVs) to AUVs and buoys to AUVs. A complete underwater wireless communication network could be deployed through various configurations involving floating buoys, AUVs, remotely operated vehicles, marine sensors, submarines, and ships. In addition, floating devices equipped with sensors can monitor ocean dynamics and collect valuable data. Among the parameters that can be obtained include the variation in the ocean surface height and direction as a function of the wind speed [4]. Wave height and direction angle data could be used to predict the behavior of the ocean surface, providing critical insights into the initial design considerations and operational strategies for NLOS-UOWC links. By integrating this information with advanced pointing and tracking mechanisms, communication nodes can improve the feasibility and performance of underwater optical links, ensuring more reliable connectivity in dynamic marine environments. On the other hand, it is worth noting that if the optimal surface reflection point configured for the communication link becomes inaccessible due to obstacles such as ships or other similar objects, the proposed model demonstrates that it is possible to take advantage of different regions or windows of coverage. Intermittent communication opportunities, which appear due to the variations in the ocean surface, would not exist if the ocean–air interface were flat. Therefore, even if the light beam does not directly impinge on the defined optimum point, the displacement of the reflected beam on the receiver plane could make it possible to reach the receiver node, thus ensuring the possibility of transmitting information. In this context, a comprehensive underwater optical channel model is crucial for overcoming the inherent challenges of the ocean environment. Key phenomena that cause a loss of intensity or the alteration of the optical beam’s direction—such as absorption and scattering—must be carefully analyzed. Additionally, the impact of the surface roughness at the ocean–air interface is significant, as it affects the reflection, refraction, and scattering of optical signals. Understanding these factors is essential for accurately modeling and improving underwater optical communication systems.

The study of underwater optical wireless communication systems is very diverse. The studies conducted cover different approaches, including practical ones. Some studies highlight the importance of realistic channel models in optimizing the UOWC system performance for diverse underwater environments. In [5], the authors employed an empirical channel model to derive closed-form expressions for metrics such as the bit error rate (BER) and outage probability, highlighting the interaction between air bubble-induced fading and scattering effects in turbid waters. As an alternative approach, in [6], the authors analyzed the role of photodiode arrays in enhancing the performance of UOWC systems, mainly focusing on diffused line-of-sight (DLOS) and NLOS configurations. The study proposed a model to estimate the received power distribution, addressing the challenges of irregular intensity distributions in mobile UOWC links. The research highlights the importance of dynamically shape-changing arrays to maintain robust communication under varying alignment conditions. In addition to this, to highlights the potential of UWOC systems to achieve high data rates and robust communication links in underwater environments, in [7], the authors investigated the propagation of optical signals through different types of Jerlov water, utilizing light-emitting diodes (LEDs) as transmitters. They explored modulation techniques, including 8-PPM and 256-PPM, to assess their effectiveness in line-of-sight (LoS) and NLOS scenarios.

An underwater optical wireless channel model with an NLOS configuration relies heavily on the characteristics of the ocean–air interface. As a result, the accuracy of the model is significantly influenced by the condition of the ocean surface. Consequently, several channel models for NLOS links have been proposed in the literature [8,9] (and the references therein). The study in [8] examined an NLOS scattering channel for underwater optical wireless communication within the optical transmission window (wavelengths between 400 nm and 600 nm). The channel was modeled using the Monte Carlo ray tracing technique, allowing for a detailed calculation and analysis of the channel impulse response (CIR) and propagation losses. Meanwhile, in [9], the propagation losses were calculated for an NLOS link following a geometrical analysis of the channel characteristics. Here, the studied scenarios considered laser diodes (LDs) and light-emitting diodes (LEDs) as optical sources, as well as two types of water: clear ocean and coastal waters. These studies assumed the ocean–air interface to be a smooth surface, acting as a perfect mirror for analyzing the underwater optical channel. The assumption of this type of marine surface has significant implications for the modeling and performance evaluation of UOWC systems [10,11,12].

However, in a more realistic scenario, the sea surface is constantly changing due to, among other factors, the wind speed and atmospheric conditions, which translate into ocean currents. Thus, natural ocean waves experience constant changes in their normal state (calm sea). The natural movement of air at different velocities and the weather conditions generate complex variations of distinct intensities in the roughness of the sea surface. Similarly, moving ocean wave patterns generate multiple reflections and refractions of light beams, which impinge on the ocean–air interface. The main elements of the sea surface that determine its roughness are the surface slopes and the increase in the area of the wind-roughened surface compared to the area of the calm ocean [13]. Various studies have been conducted in the scientific literature on NLOS-UOWC systems in which the sea surface is considered rough or wavy. In general, the current literature evidences a higher degradation in the stability and reliability of the established optical communication links concerning the performance shown when the system considers a smooth sea surface. In [14,15], the authors evaluated the bit error rate (BER) performance under a random sea surface that incorporated the effect of the wind speed to model the optical channel. Here, the results showed that a wavy surface can reduce the performance of an NLOS-UOWC system compared to a flat surface. In [16], a capillary wave rough sea surface model was considered, and a multiple channel model was presented using the Monte Carlo ray tracing approach. The authors evaluated the communication system using the path loss (PL) and the channel impulse response (CIR). In [17], an optical model based on the numerical simulations method was developed. In [18], the authors optimized Monte Carlo Integration (MCI) models for multiple scattering in NLOS-UOWC. They introduced improved sampling methods, with partial importance sampling increasing the computational efficiency by up to 12 times more than that of traditional models. Moreover, the influence of a wavy surface, the range, water properties, and receiver parameters were evaluated. The results confirmed that a wavy surface can cause remarkable reductions in the system performance. However, the referenced studies did not consider the variable size of the footprint of the optical beam reflected by the wavy surface on the plane of the receiving node. In addition, they overlooked the possibility that the link could be intermittent, as the photo-detector is only illuminated by the reflected light beam at certain times of the potential communication. In summary, the proposed model in this study performs an exhaustive deterministic geometrical analysis of an underwater optical wireless communication system. Unlike other studies, the deterministic approach ensures that no model parameters for a wavy surface assume random values. Furthermore, the Gaussian source considered in the study is characterized by its incident footprint on the ocean surface. The results showed variability in both the footprint size and the position of the beam reflected towards the incident medium. The methods presented in the literature generally focused on quantifying or calculating the receiving probability of the photons detected by the receiver node after propagation through the underwater channel, ignoring the natural behavior of the ocean–air interface. The proposed model also incorporates propagation losses when the incident ray does not satisfy the total internal reflection phenomenon due to the inherent motion of the ocean surface. Therefore, the results of these studies suggest the importance of further research and contributions to the study of NLOS-UOWC systems, taking into account more accurately and realistically the changing characteristics of the ocean surface.

In this paper, we introduce a novel NLOS-UOWC channel model that accounts for the complex dynamics of a wavy ocean–air interface. Our model delivers a precise and thorough analysis of how ocean waves affect the underwater communication system, going beyond previous studies. One of the standout contributions of this work is its ability to accurately determine the random size of the beam footprint as it reflects off the ocean surface. Additionally, we precisely tracked the moments and locations where the optical beam struck the wave surface, critical factors that define the duration of receiver illumination. For the first time, our findings demonstrate that the variability of the ocean surface causes misalignment in the NLOS-UOWC system, leading to intermittent but exploitable windows for effective communication. This study offers essential insights that push the boundaries of underwater optical communication.

The remainder of this article is organized as follows. The configuration of the proposed NLOS-UOWC system model, which considers a linear superposition of *N* sinusoidal waves to represent a wavy surface, is described in Section 2. In Section 3, the NLOS-UOWC channel model is described in detail. The numerical results and discussions are presented and analyzed in Section 4 and Section 5, respectively. Finally, in Section 6, we summarize the main conclusions and some directions for the future of this work.

## 2. Problem Definition

**Figure 1:**
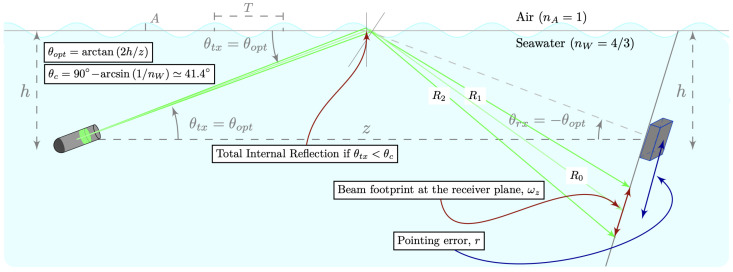
NLOS-UOWC system configuration.

The proposed NLOS-UOWC system configuration is illustrated in Figure 1. In this setup, both nodes, the transmitter (Tx) and receiver (Rx), are located at a depth of *h* meters below the perfectly flat ocean surface, represented in Figure 1 by a horizontal gray-colored line. A distance of *z* meters horizontally separates both nodes. Meanwhile, a single wave with the amplitude *A* and spatial period *T* is initially considered to model the marine surface.

The projections of the reflected rays $R_{0}$, $R_{1}$, and $R_{2}$ are also represented, with $R_{0}$ being the central ray of the reflected beam. In Figure 1, the pointing error, *r*, is the offset from the center of the receiver to the center of the reflected beam’s footprint on the receiver plane.

Furthermore, the theoretical framework for a reflective NLOS configuration in a UOWC system indicates that for the light beam generated by the transmitter node to reach the receiver, it must propagate upwards toward the ocean–air interface. Thus, in this study, the light beam was considered to be delimited by two rays, and the transmission angle, $\theta_{tx}$, was the angle between the center of the beam emitted towards the ocean surface and the horizontal plane that joins both nodes.

Consequently, in an ideal scenario, assuming that the optical source transmitted a single infinitely narrow beam, i.e., an ideal optical source with no divergence, the optimal pointing angle, $\theta_{opt}$, toward a perfectly flat reference surface would be the only transmission angle, for which the receiver node would collect the highest amount of reflected light energy, so that $\theta_{tx}=\theta_{opt}$.

Thereby, both the transmitter and receiver nodes define their pointing angles, $\theta_{tx}$ and $\theta_{rx}$, respectively, for the horizontal reference plane between both nodes, being satisfied that

(1) $$\theta_{rx}=-\theta_{opt}.$$

On the other hand, the theoretical framework for NLOS reflective links states that to maximize the amount of light received with the minimum power loss at the Rx end node and, hence, the efficiency of communication, the principle of the total internal reflection (TIR) phenomenon should be satisfied. According to the second Snell’s law, TIR occurs at the ocean–air interface when the optical signal traveling from the incident medium to the air strikes the ocean surface with $\theta_{tx}<\theta_{c}$, where $\theta_{c}$ is the critical angle.

Similarly, by applying fundamental algebraic transformations and taking into account the TIR condition described above, the relationship between *z* and *h* needed for the TIR phenomenon to occur is expressed as

(2) $$z⩾h\left(\frac{2}{\tan(\theta_{c})}\right).$$

This expression highlights the restriction of possible NLOS link configurations as a function of the relationship between the distance nodes and their depth. It should also be noted that additional losses may appear due to refraction at the ocean surface, which may further affect the overall system performance.

In a more realistic scenario, an NLOS-UOWC system will be affected by both sea surface variability caused by the dynamic influence of the wind and the optical properties of the water. Thus, sea surface waves are not static but in constant motion, continuously changing their amplitude, frequency, and direction over time. These temporal variations introduce additional challenges for the optical signal in marine environments, which can result in fluctuations and distortions in the received signal [10,19]. Besides, these variations in the wave patterns and surface roughness could impact the reflection phenomena at the ocean–air interface, causing the optical signal to deviate from the projected path [20]. As a consequence, this could cause misalignment errors in the communication system [21].

In addition, several studies have been conducted in the available literature considering the relationship between the wind speed and sea waves [22,23,24]. Further, the relationship between the ocean surface roughness, wave height, and steepness has also been studied in [25]. In [26], the authors estimated numerical values for the wave heights *A* and the period *T* (or length) of any combination of the wind velocity, fetch, and duration. In this paper, we assume that the wave height is the vertical distance from the crest (maximum height) or valley (minimum height) to the still water level, as illustrated in Figure 1.

**Figure 2:**
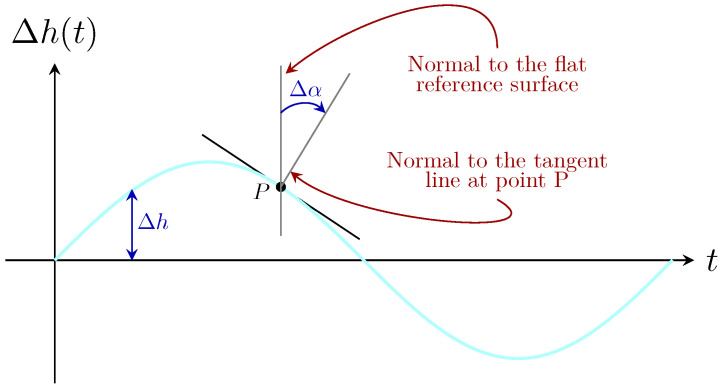
Vertical cross-section of ocean water with a sinusoidal wave-shaped surface, representing the simplest form of wave modeling.

Meanwhile, the incidence point of the optical beam on the ocean surface, denoted as *P* in Figure 2, is influenced by the wave height variations, ∆h, and changes in the angle of the local normal to ∆h(t) at that point, represented as ∆α. Therefore, in order to determine the precise location of the light beam on the wavy surface and its posterior reflection towards the incident medium, we considered the point of incidence *P* at time instant *t*, shown in Figure 2. The continuous variation in the height of the sea surface, ∆h, as well as the variation in the angle of the local normal, ∆α, cause the reflected rays forming the optical beam to shift their position on the receiver plane.

Therefore, the inherent optical properties (IOPs) of ocean water are also essential to the performance of the NLOS-UOWC system. The propagation of the optical beam can be significantly affected by factors such as the turbidity, suspended particles, dissolved organic matter, and the presence of various components such as phytoplankton [27]. Absorption and scattering processes cause the intensity attenuation and directional deviation of photons as they propagate through the underwater channel [28]. In addition, turbulence causes the underwater temperature and salinity to fluctuate. Then, it causes a random change in the refractive index of the transmission medium, which is the leading cause of optical signal fading. Ocean turbulence causes scintillation and waveform distortion [1,2]. In this study, we neglected the effect of turbulence since we focused on the deterministic characterization of the channel and the study of the coverage intervals due to the surface variation. Random phenomena introduced by turbulence in the irradiance were not considered. So, for the above reasons, these phenomena directly affect the transmission of the optical beam in the marine medium, causing complex variations in the energy transmitted to the surface and the subsequent reflection back to the receiver.

Thus, we considered the sea surface according to linear wave theory (or airy wave theory), which assumes that the wave amplitude *A* is small (compared with the depth *h*); despite its fundamental assumption of small wave amplitudes, it has demonstrated remarkable effectiveness in characterizing and predicting the behavior of ocean waves under various conditions [10]. However, to avoid the limitations of the wave amplitude in linear wave theory, many areas of physics expect ocean waves to be represented using a spectral or Fourier model. Under this approach, the water surface is approximated by the linear superposition of *N* sinusoidal waves, as defined by [29].

(3) $$f\left(t\right)=\sum_{i=1}^{N}A_{i}\sin\left(\frac{2\pi }{T_{i}}t+\phi_{i}\right),$$

where $A_{i}$ is the amplitude of each sinusoidal component of the wave, $T_{i}$ is the period, and $\phi_{i}$ the relative phase of the *i*th component in the summation.

## 3. NLOS-UOWC Channel Model

The UOWC channel, based on an NLOS configuration, was modeled as a single loss coefficient, *L*, expressed as

(4) $$L=h_{a}\times h_{p},$$

where $h_{a}$ is the path loss and $h_{p}$ represents the loss effects of the geometric spread and angular pointing errors of a laser optical signal due to the variability of the sea surface. The loss coefficient allows us to calculate the received optical power $P_{Rx}$ from the transmitted power $P_{Tx}$ as

(5) $$P_{Rx}=L\times P_{Tx}.$$

### 3.1. Oceanic Path Loss

Seawater is rich in various dissolved and suspended elements in different concentrations. It also contains chlorophyll, the pigment that phytoplankton use to absorb sunlight and convert it into biomass. This composition activates two physical processes that contribute significantly to the degradation of the transmitted optical signal along the light path, namely absorption, a(λ), and scattering, b(λ), which are affected by both the wavelength, λ, and the propagation distance, *d* [30]. These two phenomena cause the attenuation of the optical signal intensity and changes in the direction of propagation through the seawater channel. Hence, the effects of absorption and scattering can be calculated using the extinction coefficient, c(λ), as follows [31]:

(6) c(λ)=a(λ)+b(λ).

In Table 1, the values of a(λ) and b(λ) for clear ocean and coastal waters are summarized when considering a wavelength, λ, of 532 nm. These values correspond to a chlorophyll concentration of 0.31 mg/m^3^ for clear ocean water and 0.83 mg/m^3^ for coastal water [32].

In this way, the well-known exponential Beer–Lambert law makes it possible to calculate the propagation losses when a beam of light propagates through the ocean, as follows:

(7) $$h_{a}=e^{-c\left(\lambda \right)R_{t}d},$$

where *d* is the total propagation distance. Here, from Figure 1, *d* is defined according to a complex geometrical analysis, as follows:

(8) $$d=\frac{h+∆h}{\sin(\theta_{tx})}+\frac{B}{\cos(\beta )},$$

where ∆h is the wave height variation, and *B* and β will be defined in detail later, in Appendix A.

Meanwhile, when light impinges on an interface between two media with different refraction indexes, it experiences partial transmission/refraction and partial reflection, described through the Fresnel coefficients. In addition, as is well known, a light beam loses its polarization while propagating through a scattering medium such as an underwater environment. Therefore, it can be assumed that the light reaching the ocean–air interface is unpolarized [3,34,35].

Within this framework, it is essential to take into account the power losses associated with the reflected optical beam at the sea surface. The Fresnel reflectance coefficient, $R_{t}$, is used for this analysis. $R_{t}$ estimates the amount of light reflected to the incident medium when the total internal reflection condition is unmet. Therefore, the expression to calculate the Fresnel reflectance coefficient is given by

(9) $$R_{t}=\frac{R_{p}+R_{ll}}{2},$$

where $R_{p}$ and $R_{ll}$ are the reflectances for perpendicular and parallel polarized light, respectively. Likewise, $R_{p}$ and $R_{ll}$ are given by the following:

(10) $$R_{p}={\left|\frac{n_{W}\cos\left(\theta_{i}\right)-n_{A}\cos\left(\theta_{tx}\right)}{n_{W}\cos\left(\theta_{i}\right)+n_{A}\cos\left(\theta_{tx}\right)}\right|}^{2},$$

(11) $$R_{ll}={\left|\frac{n_{W}\cos\left(\theta_{tx}\right)-n_{A}\cos\left(\theta_{i}\right)}{n_{W}\cos\left(\theta_{tx}\right)+n_{A}\cos\left(\theta_{i}\right)}\right|}^{2},$$

where $\theta_{i}$ and $\theta_{tx}$ are the incident and transmission angles, respectively. The derivation of the angle of incidence, $\theta_{i}$, of the reflection on the sea surface depends on the parameters that define the shape of the wave and the link configuration parameters, such as the depth *h*, the separation between nodes *z*, and the angle at which the transmitter points to the ocean surface to reach the receiver, $\theta_{tx}$. This analysis is non-trivial, requiring a trigonometric development detailed in Appendix A.

### 3.2. Geometric Loss

The dynamic nature of sea waves represents a major challenge in UOWC links based on an NLOS configuration, where the sea surface serves as a reflection medium to overcome obstacles during data transmission. The constant movement and deformation of the water surface due to waves introduce unavoidable fluctuations on the receiver plane, leading to an inherent pointing error never before explored in NLOS-UOWC channel modeling. This pointing error occurs as the reflection angle at the ocean–air interface continuously changes, causing the reflected optical beam to shift its unpredictable position relative to the receiver. In addition, this error can cause momentary misalignment between the transmitter and receiver, severely impacting the quality and reliability of the communication link. To model the inherent pointing error between the transmitter and receiver, we used the model proposed in [36], widely used in the scientific literature, in which the effects of the beam width on the receiver plane, $\omega_{z}$, the beam divergence angle, $\theta_{div}$, and the diameter of the circular photo-detector aperture, *D*, are taken into account.

The geometric spreading attenuation at the receiver, considering a pointing error, *r*, was analytically modeled for a laser beam propagating through seawater. Specifically, the off-axis received power was calculated for a laser source with a Gaussian intensity profile, where the beam center was displaced by a distance, *r*, from the center of a circular receiver aperture with the diameter *D* [37].

Thus, the attenuation due to the geometric spread, $h_{p}$, is given by

(12) $$h_{p}\approx A_{0}\exp\left(-\frac{2r^{2}}{\omega_{z_{eq}}^{2}}\right),$$

where $\omega_{z_{eq}}^{2}$ is the equivalent beam width in the receiver and is represented as

(13) $$\omega_{z_{eq}}^{2}=\omega_{z}^{2}\frac{\sqrt{\pi }\mathrm{erf}\left(v\right)}{2v\exp(-v^{2})},$$

where

(14) $$v=\frac{\sqrt{\pi }D}{\sqrt{2}\omega_{z}},$$

where *D* is the receiver aperture diameter and $A_{0}$ is the fraction of the collected power at r=0, i.e., when the central ray $R_{0}$ strikes the center of the receiver, and is given by

(15) $$A_{0}={\left[\mathrm{erf}\left(v\right)\right]}^{2},$$

with erf(·) being the error function.

### 3.3. Angular Pointing Error Analysis

As previously mentioned, as the optical beam is projected towards the sea surface, a beam footprint is generated at the ocean–air interface, which is affected by continuous ∆h and ∆α variations, as shown in Figure 2. Meanwhile, when total internal reflection occurs, a portion of the energy radiated by the transmitter will return to the incident medium and occasionally illuminate the receiver. In this regard, the offsets $r_{1}$ and $r_{2}$, measured from the center of the photo-detector to the intersection point of the reflected rays $R_{1}$ and $R_{2}$ with the receiver plane, respectively, represent the footprint of the optical beam reflected to the incident medium, as shown in Figure 1.

Thereby, through complex trigonometric operations, it was found that the pointing error, $r_{i}$, associated with the reflected ray $R_{i}$, as detailed in Appendix A, can be expressed as follows:

(16) $$r_{i}=\sqrt{\xi -\sigma }.$$

Following the calculation of the values of $r_{1}$ and $r_{2}$ using the provided expressions in Appendix A, the pointing error *r* can be computed as

(17) $$r=\frac{r_{1}+r_{2}}{2}.$$

Equivalently, the width of the reflected beam on the receiving plane, $\omega_{z}$, is given by

(18) $$\omega_{z}=\left|r_{1}-r_{2}\right|.$$

## 4. Results

In this section, the numerical results are presented for two different types of water, considering clear ocean and coastal waters and a separation between the transmitter and receiver of z=10 m when both nodes are at a depth of h=4 m with respect to the average level of the ocean surface. Moreover, a receiver with a nominal diameter of D=10 cm and a field of view of 180° was also assumed, as well as a laser source operating at a wavelength of λ=520 nm with a divergence angle of $\theta_{div}=15$ mrad. The main NLOS-UOWC system parameters taken into account here are summarized in Table 2.

**Figure 3:**
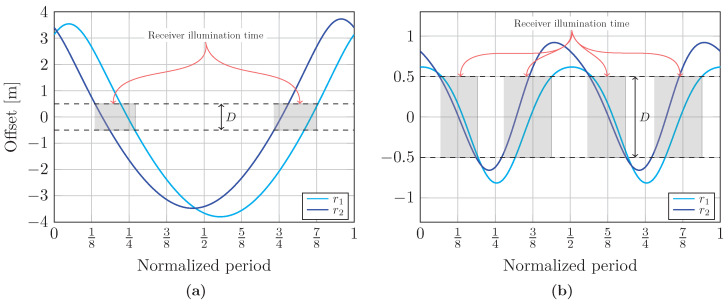
The offsets, $r_{1}$ and $r_{2}$, from the center of the receiver to the intersection of the rays $R_{1}$ and $R_{2}$ with the receiver plane, respectively, when considering (**a**) a simple wave and (**b**) a complex wave, where the shaded area corresponds to the time for which the receiver remains illuminated concerning the period, *T*, of each wave.

**Figure 4:**
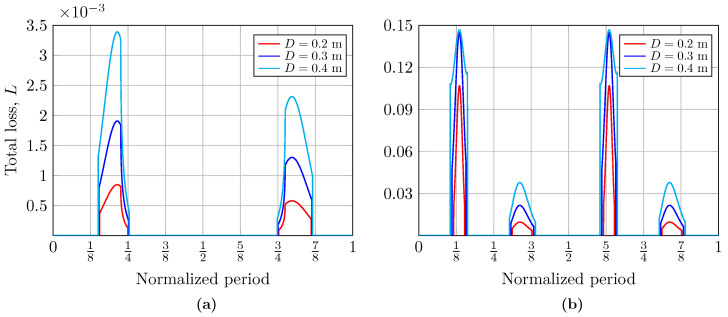
Total loss coefficient, *L*, for different receiver aperture diameters and clear ocean water when considering (**a**) a simple wave and (**b**) a complex wave.

**Figure 5:**
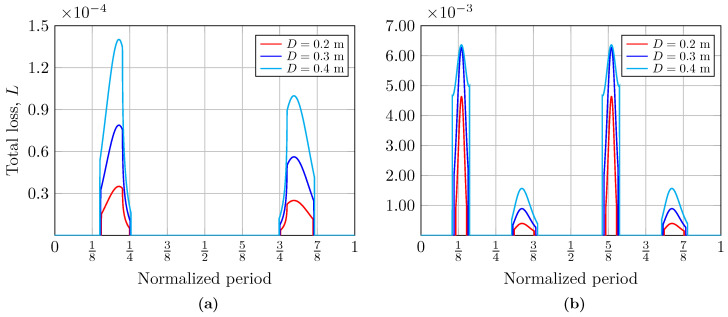
Total loss coefficient, *L*, for different receiver aperture diameters and coastal water when considering (**a**) a simple wave and (**b**) a complex wave.

**Figure 6:**
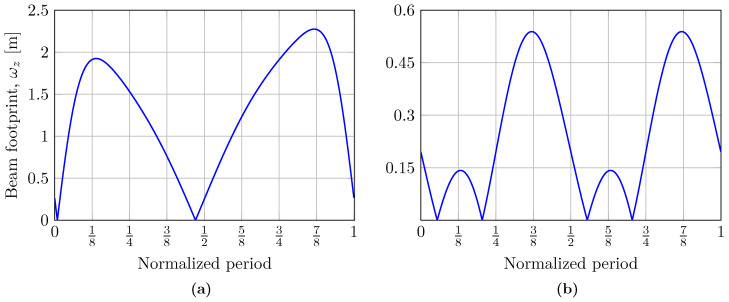
Beam width of the reflected optical source, $\omega_{z}$, as a function of the period of the wave, considering (**a**) a simple wave and (**b**) a complex wave.

In general terms, Figure 3 shows the offsets $r_{1}$ and $r_{2}$ from the center of the receiver to the intersection of the $R_{1}$ and $R_{2}$ rays with the receiver plane, as well as the time percentage for which the receiver node remains illuminated concerning the period, *T*, of the considered wave. Putting it another way, Figure 4 and Figure 5 show the total loss coefficients, *L*, for different receiver node aperture diameters, analyzed for two water types: clear ocean and coastal waters, respectively. Finally, the beam width of the reflected optical source, $\omega_{z}$, as a function of the period of the wave is shown in Figure 6. It is important to note that the results considering two different types of water are presented only for the calculation of the loss coefficient, *L*, since the computation of the offsets, $r_{1}$ and $r_{2}$, as well as that of the variable size of the footprint, $\omega_{z}$, does not depend on the type of water. It should be noted that all the results were analyzed considering two types of ocean waves: (a) a simple wave (N=1) using a sinusoidal wave with $A_{1}=0.07$ m and $f_{1}=0.6$ Hz, and (b) a complex wave (N=2) determined by summing two sinusoidal waves with $A_{1}=0.17$ m, $A_{2}=0.25$ m, $f_{1}=0.2$ Hz, and $f_{2}=0.1$ Hz [26].

## 5. Discussion

This section presents a comprehensive analysis of the receiver node’s illumination time at the moments when the reflected beam reaches it. Similarly, the behavior of the total loss coefficient, *L*, as calculated in this study, is evaluated. In addition, the variation in the beam size, $\omega_{z}$, on the receiver plane is analyzed.

### 5.1. Receiver Illumination Time Analysis

As discussed earlier, $r_{1}$ and $r_{2}$ represent the offsets from the center of the receiver node to the intersection of the $R_{1}$ and $R_{2}$ rays with the receiver plane, respectively. In Figure 3, these offsets are plotted as a function of the period, *T*, for both the simple and complex waves. This analysis revealed whether the receiver captured the optical power and for how long. In addition, in both figures, the two horizontal dashed lines perpendicular to the ordinate axis represent the diameter of the receiver aperture. Note that, for representation purposes, an academic diameter has been used for both plots to define the percentage of the receiver node’s illumination time concerning the wave period. This percentage corresponds to the area defined by the intersections of $r_{1}$ and $r_{2}$, with the dashed lines representing the diameter of the receiver. In Figure 3, for the case of a simple wave, the percentages of the receiver’s illumination time concerning the wave period are $\%I_{time}=\left\{20.27,\;21.25,\;22.16\right\}$% when considering receiver aperture diameters of D={0.2, 0.3, 0.4} meters, whereas for the case of a complex wave, the receiver’s illumination times relative to the wave period are $\%I_{time}=\left\{20.5,\;24.58,\;28.83\right\}$% when assuming the same diameter sizes for the receiver.

From the analysis performed, it can be inferred that under these conditions, NLOS UOWC links tend to be intermittent. In addition, the bit rate achieved during the receiver’s illumination time will directly depend on the total bit rate considered for the link, which will determine whether this is sufficient to guarantee the reliability of the communication system.

### 5.2. Total Loss Coefficient Analysis

In Figure 4 and Figure 5, we present the computed total loss coefficients, *L*, as a function of the normalized wave period during the periods in which the receiver is illuminated by the beam reflected by the sea surface, i.e., when the communication system has coverage. Hence, to evaluate the impact of the area of the receiver aperture, the total loss coefficient was calculated for different diameters of D={0.2, 0.3, 0.4} meters. In Figure 4 and Figure 5, the curves represent the moments in the period of a single wave at which the receiver is illuminated, thus receiving optical power. The curves on the left correspond to the crest of the wave, while those on the right correspond to its valley. The percentage of time for which the crest and the valley of the wave are illuminated is asymmetric, as can be seen; this explains the scaling of the curves. In addition, under these link conditions, the influence of the size of the aperture diameter of the receiver is evident: an increase in its size improves the received power, which can be observed in the widening of the obtained curves. Likewise, the total losses at the receiver node when the beam is incident on both the crest and valley when considering a complex wave are also shown in Figure 4. Both graphs also allow us to analyze how the Fresnel coefficients influence the calculation of total losses, depending on whether or not the total internal reflection phenomenon is fulfilled. The influence of these coefficients will depend on the link parameters. Their impact tends to vary the loss curves irregularly.

Meanwhile, the scaling of the total loss coefficient curves in Figure 5 concerning Figure 4 is evident. The high concentration of suspended sediments, plankton, and dissolved organic matter in coastal water reduces its clarity and increases the turbidity, thus increasing the absorption and scattering effect, decreasing the light beam intensity, and limiting communication.

### 5.3. Beam Width Analysis

In Figure 6, the variation in the beam width, $\omega_{z}$, on the receiver plane as a function of the wave period, *T*, is shown. It is worth mentioning that this beam width varies due to the direct impact of sea waves, a novel feature compared to underwater optical wireless communication links in an LOS configuration, where the beam width remains constant. It follows that the relationship between the beam width and the divergence of the laser source, $\theta_{div}$, is crucial. Therefore, an increase in $\theta_{div}$ results in a wider reflected beam width or beam footprint on the receiver plane, consequently reducing the received optical power. On the other hand, these findings provide valuable insight into the beam width variability in hostile underwater environments and can facilitate the performance of statistical analyses considering different, more complex sea surface conditions.

## 6. Conclusions

This paper represents a significant contribution to the detailed and novel understanding of how sea waves influence NLOS-UOWC systems. By adopting a deterministic wave approach, the research highlights the crucial role of the sea surface variability in affecting both the beam width and its position on the receiver plane. This allows for optimized transmission during moments when the receiver is properly illuminated. These findings can serve as a solid foundation for refining the design of underwater optical wireless communication systems that rely on an NLOS configuration. Despite the deterministic approach, this study opens the door for future research that incorporates a stochastic analysis of sea wave behavior. Such an approach would provide a deeper insight into how random wave fluctuations impact the system performance, particularly in terms of the BER. This next step is essential for enhancing the reliability and robustness of NLOS-UOWC systems in real-world oceanic environments. Future research will explore the integration of the proposed model with AI-based approaches, such as machine learning models, to allow it to dynamically adapt to real-time environmental changes and enhance the system performance in practical scenarios. In addition, future experimental validation in a controlled water tank environment will investigate the effects of real-world factors such as turbulence and varying wave conditions, thereby refining and extending the proposed model.

## Figures and Tables

**Table 1:** Typical values of absorption, a(λ), scattering, b(λ), and extinction, c(λ), coefficients for different water types [33].

Water Type	$a[m^{-1}]$	$b[m^{-1}]$	$c[m^{-1}]$
Clear ocean water	0.114	0.037	0.151
Coastal water	0.179	0.219	0.398

**Table 2:** NLOS-UOWC system parameters.

Parameter	Symbol	Value
Wavelength	λ	520 nm
NLOS-UOWC link distance	*z*	10 m
NLOS-UOWC link depth	*h*	4 m
LD divergence angle at 1/e	$\theta_{div}$	15 mrad
Photo-detector aperture diameter	*D*	10 cm
Receiver field of view	FOV	180°

**Figure A1:**
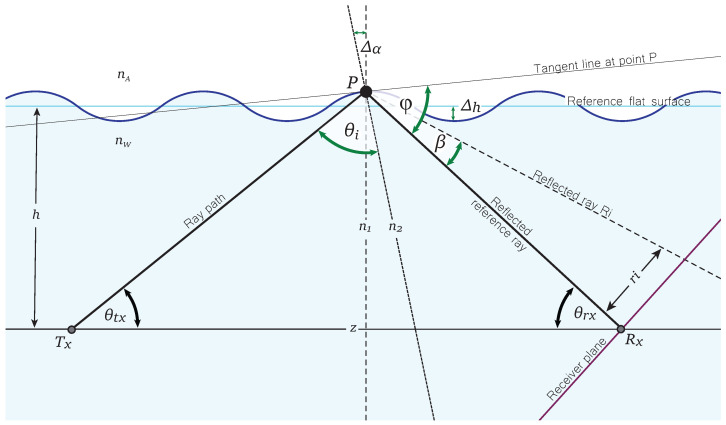
Geometric model for analyzing and estimating parameters critical to calculating pointing errors.

## Data Availability

The data will be made available on request.

## Appendix A. Angular Pointing Error Modeling

Figure A1 illustrates the geometrical model proposed to analyze the impact of the ocean surface on the incident light beam generated by the transmitter node upon reaching the ocean–air interface and its subsequent reflection towards the receiver node. In this configuration, the transmitter and receiver nodes are oriented towards the ocean surface at point *P*, with their respective transmission and reception angles denoted as $\theta_{tx}$ and $\theta_{rx}$.

The surface roughness at *P* introduces a local normal, $n_{2}$, which deviates by ${∆}_{\alpha }$ degrees from the normal to the flat reference surface, $n_{1}$. Consequently, the angle of incidence, $\theta_{i}$, is set between the incident ray and $n_{2}$, reflecting the direct influence of the wavy surface conditions.

Through a complex trigonometric analysis, the pointing error, $r_{i}$, associated with the reflected ray $R_{i}$, is modeled analytically as follows:

(A1) $$r_{i}=\sqrt{\xi -\sigma },$$

where ξ is given by

(A2) $$\xi ={\left(\frac{\sqrt{{\left(h+∆h\right)}^{2}+(z-{\left(\cot\left(\theta_{tx}\right)\left(h+∆h\right)\right)}^{2}}}{\cos(\beta )}\right)}^{2},$$

where *h* is the depth at which the nodes are located, ∆h is the wave height, *z* is the link distance, and the transmission angle is denoted as $\theta_{tx}$.

Here, β and σ are represented as

(A3) $$\beta =\theta_{i}+\varphi -\frac{\pi }{2},$$

(A4) $$\sigma =\sqrt{{\left(h+∆h\right)}^{2}+(z-{\left(\cot\left(\theta_{tx}\right)\left(h+∆h\right)\right)}^{2}},$$

where $\theta_{i}$ represents the incident angle,

(A5) $$\theta_{i}=\frac{\pi -2(\theta_{tx}-∆\alpha )}{2},$$

and

(A6) φ=arccos(γ),

with

(A7) $$\gamma =\frac{z\times \cos\left(∆\alpha \right)-\left(h+∆h/\sin\left(\theta_{tx}\right)\right)\cos\left(\theta_{tx}-∆\alpha \right)}{\sqrt{{\left(h+∆h\right)}^{2}+{\left(z-\left(\cot\left(\theta_{tx}\right)\left(h+∆h\right)\right)\right)}^{2}}},$$

where ∆α represents the local normal at the incident point of the light beam at the ocean surface.
